# A combustion method to synthesize nanoporous graphene†

**DOI:** 10.1039/c7ra13568h

**Published:** 2018-03-05

**Authors:** Q. Y. Yang, H. L. Zhou, M. T. Xie, P. P. Ma, Z. S. Zhu, W. Zhu, G. Z. Wang

**Affiliations:** Key Laboratory of Strongly-Coupled Matter Physics, Chinese Academy of Sciences, Hefei National Laboratory for Physical Science at Microscale, Department of Physics, University of Science and Technology of China Hefei Anhui 230026 P. R. China gzwang@ustc.edu.cn

## Abstract

In this paper, we introduce a combustion method which is rapid, low cost, mass-producing and environmentally friendly to produce nanoporous graphene. After loading a graphene oxide aerogel (GOA)/paper (GOP) on a preheated hot plate (as the heat source, with a temperature as low as 200 °C) under an ambient environment, in a few seconds, the GOA/GOP would self-combust and change into reduced graphene oxide (RGO) with nanopores mainly concentrated in the 0.4–2.0 nm range and a large specific surface area of 536 m^2^ g^−1^. Supercapacitors fabricated with the synthesized porous RGO (P-RGO) showed a high specific capacitance of 245 F g^−1^ at 0.1 A g^−1^, and a retention rate of about 96.9% after 12 000 cycle tests with respect to the initial specific capacitance with a scan rate of 10.0 A g^−1^. The production yield of this method was as high as 77.0%.

## Abstract 2

The combustion process of GOA, and the specific surface area and pore size distribution of P-RGO are shown in the images.

## Graphical abstract

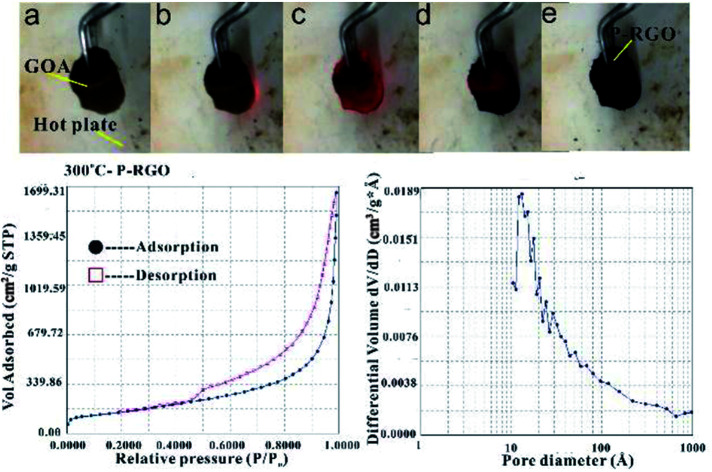

## 1. Introduction

Graphene,^1^ a one-atom-thick two-dimensional (2D) carbon material, has attracted people's attention since it was first obtained in 2004.^2^ Because of its ultra-high theoretical carrier mobility (∼200 000 cm^2^ V^−1^ s^−1^),^3^ large specific surface area (SSA) (∼2630 m^2^ g^−1^), considerable thermal conductivity (∼5000 W m^−1^ K^−1^),^4^ good electrical conductivity and optical transmittance (∼97.7%),^5^ graphene has achieved widespread application in the fields of electronic and photonic devices,^6,7^ energy storage,^8^ gas separation^9,10^ and sensors.^11,12^

However, in applications such as those involving supercapacitors, lithium ion batteries and so on, graphene sheets tend to self-aggregate irreversibly due to the strong π–π bonds and the van der Waals forces between graphene layers^13^ and even to form graphite layers, thus leading to a sharp reduction of the SSA. In addition, the self-aggregation would block the transmission of electrolyte ions, which would impede the progress of electrochemical process. To reduce the influence of the self-aggregation and fully take advantage of the intrinsic high SSA of graphene, researchers turn the attention to nanoporous graphene,^14–17^ which had porous structure, high SSA, and large enough pore volume (which benefits the transmission of electrolyte ions). During the past years, reactive ion etching methods^18–20^ have been used to make pores in the graphene sheets. These methods could produce porous graphene easily and were environment-friendly. However, the mass production of porous graphene is still a big challenge. Various methods based on the chemical etching^21,22^ and catalytic oxidation^23–31^ have been studied. With these methods nanopores as small as 0.6–5.0 nm were gained. However, it was not easy to remove the reagents introduced in the process of preparation. In addition, these ways were neither environment friendly, economical, nor mass-producing. Thermal expansion based methods,^32–34^ have also been used for mass production of nanoporous graphene. However, the methods needed high temperature, high vacuum, or special ambient condition.^35,36^ Also, these methods usually had lower production yield.

In this paper, we introduce a combustion method which is rapid, low cost, mass-producing, environment friendly and no additional fuels were added in to produce nanoporous graphene from GO. The SSA of the obtained nanoporous graphene could reach as large as 536 m^2^ g^−1^ and the corresponding pore volume was 2.6 cm^3^ g^−1^. And the material was used to fabricate supercapacitor. The specific capacitance of the fabricated supercapacitor could reach as high as 245 F g^−1^ at 0.1 A g^−1^ specific current density. The retention rate of the supercapacitor was higher than 96.8% after 12 000 cycles with respect to the initial specific capacitance, which showed the electrochemical stability of the supercapacitor.

## 2. Results and discussion

**Fig. 1:**
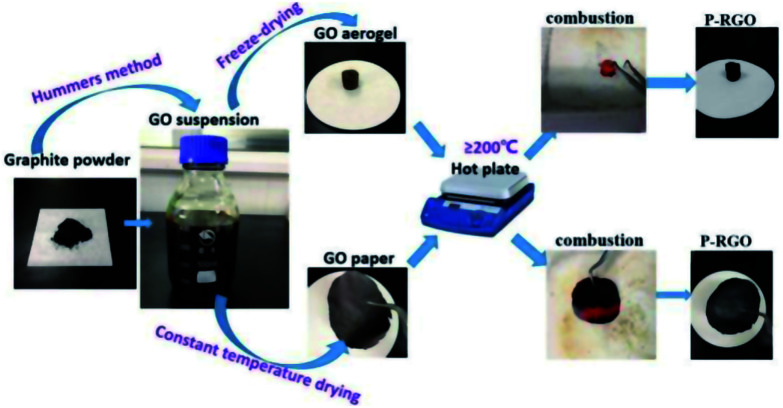
The schematic illustration displays the combustion method for preparing P-RGO. Starting from graphite powder, *via* Hummers method, GO suspension was then obtained. By way of freeze-drying/constant temperature drying, GO aerogel/GO paper was produced. Then, GO aerogel/GO paper combusted through the process of the thermal treatment, and P-RGO was obtained.

The whole process of producing P-RGO is described in the schematic diagram (Fig. 1). In this paper, we mainly discuss the sample obtained through the process with HT-300 °C (here the heat source temperature was abbreviated to HT), which was compared with the one obtained at HT-200 °C. Higher HT such as 400 °C and 500 °C led to similar results as that of HT-300 °C process. When HT was below 180 °C, the self-combustion would not happen. It would take much longer time for the occurrence of self-combustion at HT from 180 to 200 °C.

**Fig. 2:**
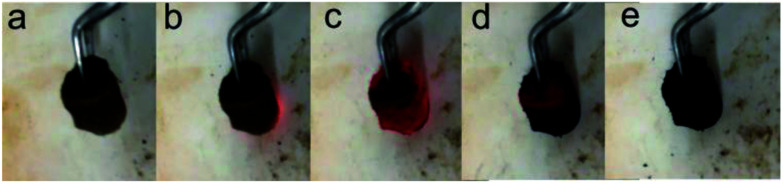
Optical photos of the combustion process of GOA. (a) The brown columnar GOA before contacting the hot plate, (b)–(d) the process of combustion in less than 0.3 s, and (e) the black fluffy P-RGO was obtained after all.

The self-combustion process of GOA on the preheated hot plate (HT-300 °C) is shown in Fig. 2a–e. (The process of GOP is shown in Fig. S.I. 1† of the ESI). When GOA contacted the hot plate, the flame immediately came up at the contacting point and then passed through the whole aerogel instantaneously (less than 0.3 s), as shown in Videos 1 and 2† in the ESI. Thus the fluffy black P-RGO was produced.

**Fig. 3:**
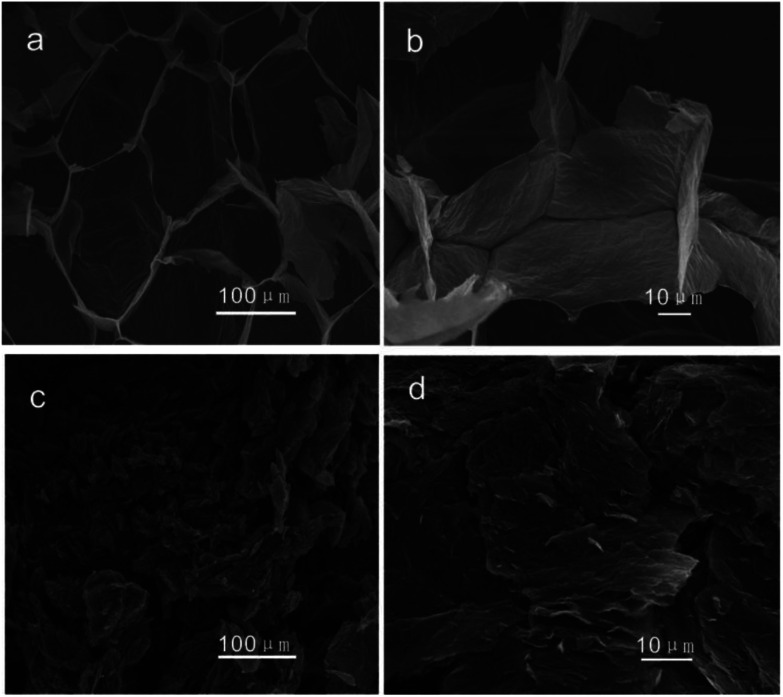
The SEM images of (a), (b) GO aerogel, and (c), and (d) HT-300-P-RGO.

Fig. 3 is the SEM images of the morphology of the GOA sample prepared by freeze-drying and the RGO sample produced by combustion. The sectional view of the GOA (Fig. 3a and b) looked like sponges. However, after the thermal treatment, GOA got reduced, expanded and broken into pieces of several micrometers in size. And the obtained material P-RGO is showed in Fig. 3c and d with visible feature: collapse, forming a loose aggregation of tiny flakes of P-RGO. From the changes of morphology and color, we could preliminarily judge that the graphene oxide had been reduced.

**Fig. 4:**
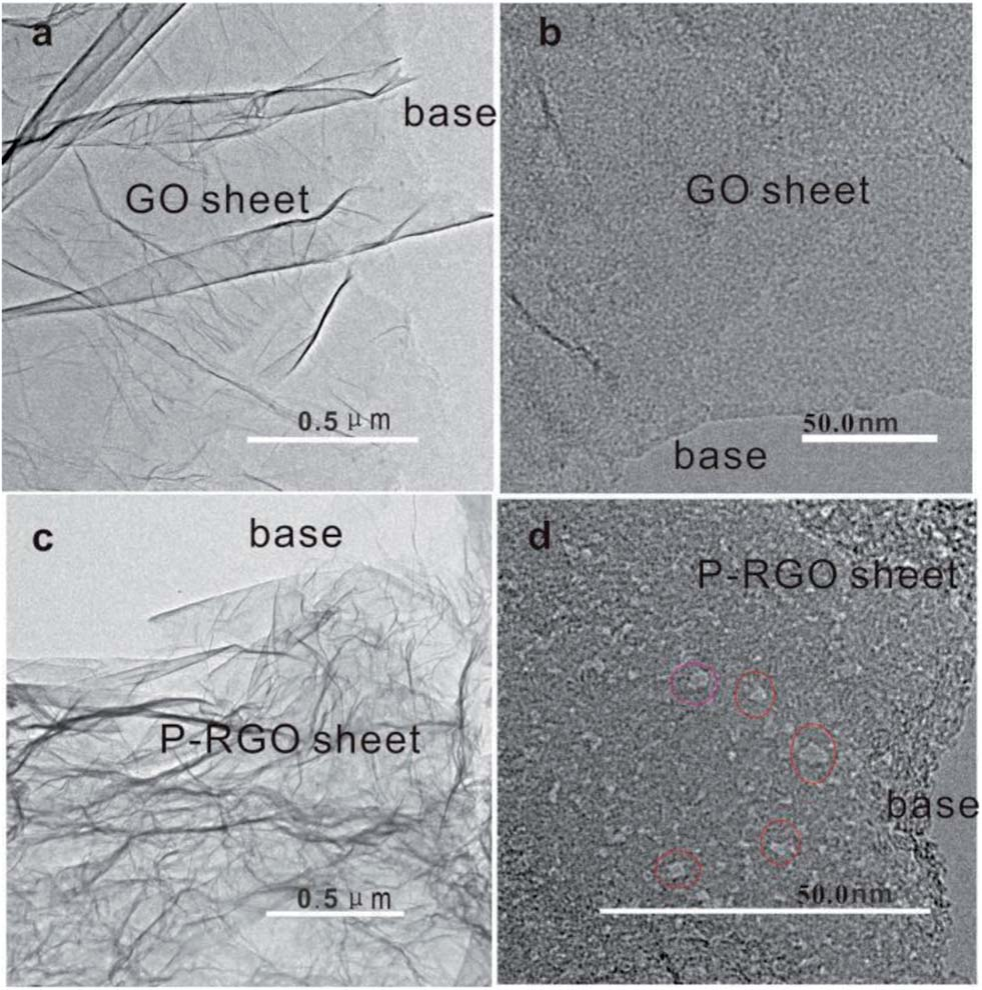
TEM images of (a, b) – GOA and (c, d) – 300-P-RGO. Red circles in (d) are used to show some nanopores.

TEM images in Fig. 4 display the surface morphology of GO and P-RGO sheet. Image (a) and (c) show the few-layer feature of the GO and P-RGO. The high-resolution TEM (HRTEM) images of the GO and P-RGO sheets in Fig. 4b and d reveal the P-RGO's porous nature. In the image (b), nearly no pores could be seen in GO sheet. However, P-RGO sheet with porous feature could be observed clearly in image (d) with some nanopores marked by red circles. Furthermore, as would be discussed later, BJH analysis indicated that the pores produced by this method were only a few nanometers in size.

**Fig. 5:**
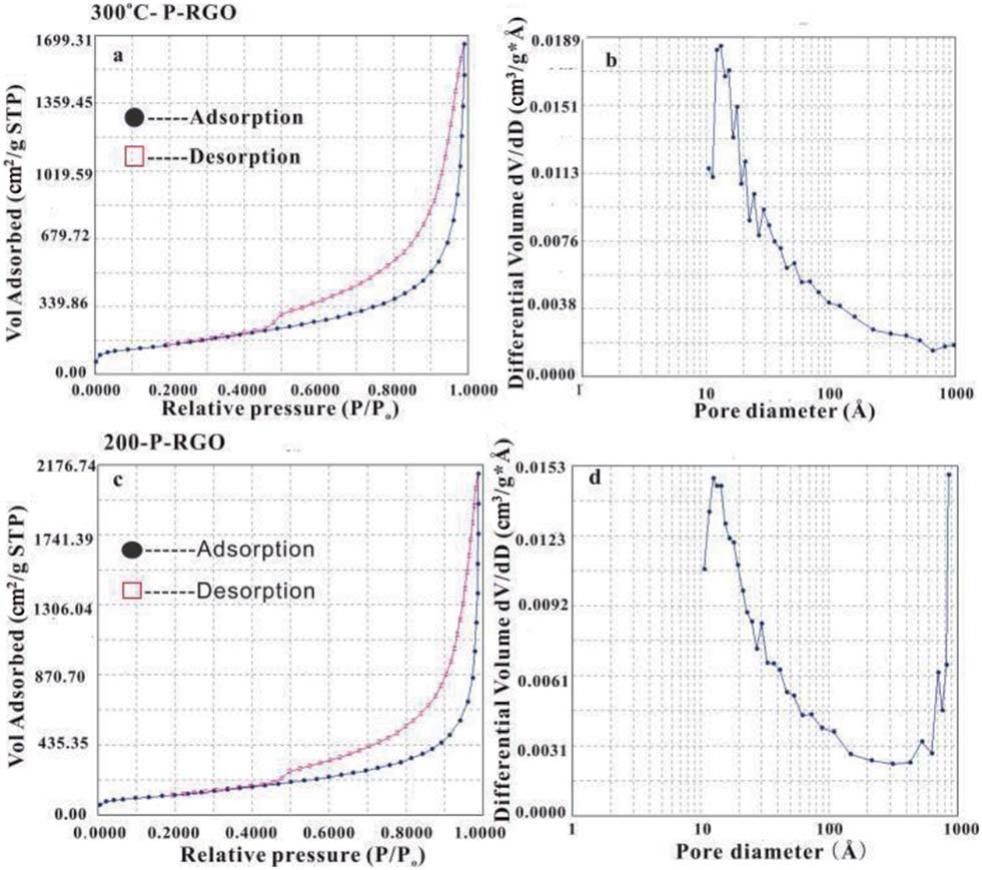
(a) N_2_ physical adsorption isotherm and (b) BJH adsorption pore size distribution of the 300-P-RGO. (c) N_2_ physical adsorption isotherm and (d) BJH adsorption pore size distribution of the 200-P-RGO.

MFA-140 was used to measure the specific surface area value by the Brunauer–Emmett–Teller (BET) method and the pore size distribution by the Barrett–Joyner–Halenda (BJH) method. The results are shown in Fig. 5 and Table 1. This measurement displayed that the 300-P-RGO sample had a relatively high SSA and a large pore volume (Fig. 5a and b), while the 200-P-RGO had a smaller SSA and a larger pore volume (Fig. 5c and d). For comparison, the results of GOA samples obtained at HT-400 °C and HT-500 °C, and GOP sample obtained at HT-200, 300 °C were also included in Table 1. As the HT increased, the SSA of obtained samples did not get larger than that of 300-P-RGO. Still, the higher temperature could not bring any superiority, which meant that HT around 200–300 °C was more proper for producing P-RGO. Fig. 5b shows the pore diameter distribution of 300-P-RGO. Clearly, the pore diameter was mainly concentrated in 0.4–2.0 nm. From the result of BJH measurement, it was known that P-RGO contained mainly micro-pores, also contained a small part of the meso-pores and macro-pores, but the contribution of meso-pores and macro-pores to SSA was very small. Importantly, in per unit volume, the greater the number of micro-pores and the smaller the diameter of the pores, then the larger the SSA would be.

**Table 1:** Specific surface area and porosity analysis of P-RGO obtained at different heat source temperatures

P-RGO (obtained at different heat source temperatures)	SSA (m^2^ g^−1^)	Pore volume (cm^3^ g^−1^)	Pore diameter distribution (Å)	Percentage of volume content of micro-pores (pore diameter ≤ 2 nm)	Percentage of volume content of meso-pores (pore diameter: 2–50 nm)	Percentage of volume content of macro-pores (pore diameter ≥ 50 nm)
200-P-RGO	457	3.3	4–856 Å	59.5%	20.5%	20.0%
300-P-RGO	536	2.6	4–970 Å	75.6%	15.4%	10.0%
400-P-RGO	452	2.7	4–830 Å	58.9%	19.8%	21.3%
500-P-RGO	498	2.6	4–917 Å	72.5%	15.8%	11.7%
200-P-RGO from GO paper	220	2.3	4–950 Å	50.1%	32.5%	17.4%
300-P-RGO from GO paper	286	1.6	4–1005 Å	66%	23.2%	10.7%

As shown in Table 1, the pore sizes of RGO prepared from GOA (HT-200 °C, 300 °C, 400 °C and 500 °C) and RGO prepared from GOP (HT-200 °C and 300 °C paper) mainly concentrated in 0.4–2.0 nm. Due to different HT, RGO had different micro-pore contents and pore diameter distributions, which led to different SSA. The RGO sample obtained at HT-300 °C had the highest SSA because of its high micro-pore contents. The reasons for lower micro-pore contents at other temperatures (HT-200 °C, 400 °C, 500 °C) can be understood as follows. For the sample obtained at HT-200 °C, the combustion process involved a relatively violent explosion, and for samples obtained at higher HT (400 °C, 500 °C) it maybe attribute to strong combustion.

**Fig. 6:**
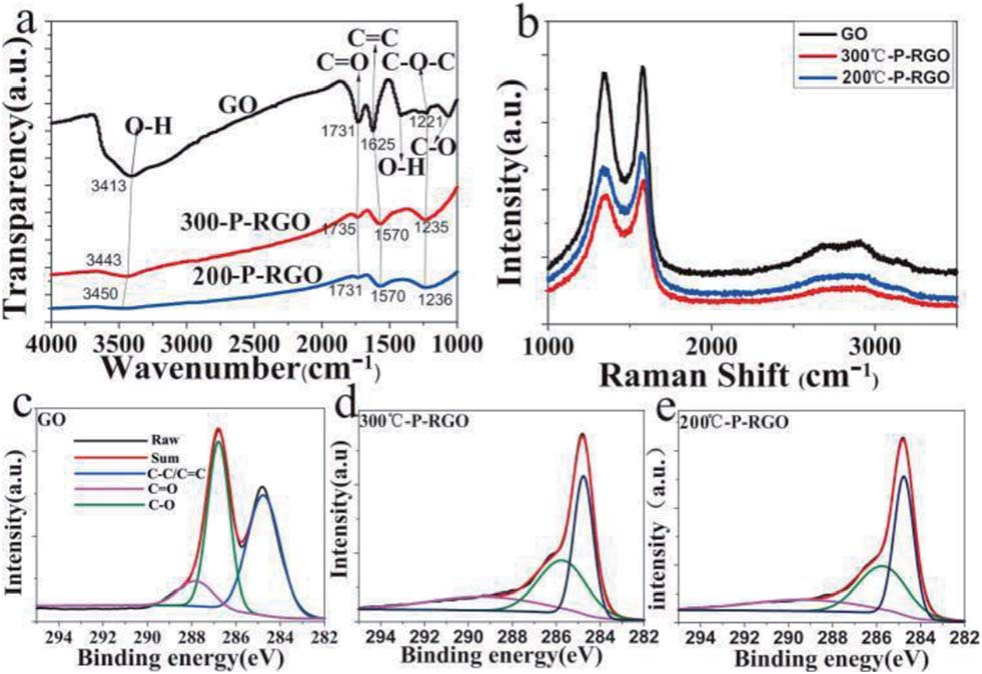
(a) FTIR spectra of GO, 300-P-RGO and 200-P-RGO samples, where the intersection points of every light grey line and the bold lines (black, red, blue) represents the same functional group; (b) Raman spectra of GO, 300-P-RGO, 200-P-RGO samples; (c–e) XPS analysis of C 1s peaks corresponding to GO, 300-P-RGO and 200-P-RGO samples, respectively.

The spectroscopy of FTIR has been studied to show whether the oxygenated functional groups were removed. As shown in Fig. 6a, the spectrum of GO sample (the black curve) demonstrated that a plenty of oxygenated functional groups were bonded to the sample. The apparently stronger and broader peaks around 3413 cm^−1^ and 1410 cm^−1^ were hydroxyl groups (O–H), the peak at 1731 cm^−1^ was carbonyl groups (C=O), the peak at 1625 could be ascribed to C=C bonds, and the peaks at 1221 cm^−1^ and 1060 cm^−1^ were C–O bonds.^35,37^ As to the P-RGO samples (HT-300 °C and 200 °C), the peaks of O–H, C–O bonds were weakened, suggesting that GO had been reduced. Clearly, the O–H bond of 200-P-GO was nearly disappeared compared to that of 300-P-RGO, which could be explained by a stronger reduction under HT-200 °C treatment condition. From the spectroscopy of FTIR, it was known that the 300-P-RGO sample had a larger oxygen content compared to the 200-P-RGO sample, which might be explained by the fact that a larger SSA of 300-P-RGO sample would adsorb more O_2_/H_2_O onto the sheets and bond with the unstable and unsaturated atoms on the surface.

Fig. 6b shows the Raman spectra of GO, 300-P-RGO, and 200-P-RGO samples, exhibiting two apparently strong G and D peaks at 1576.33 cm^−1^/1335.68 cm^−1^, 1584.00 cm^−1^/1360.21 cm^−1^, and 1570.20 cm^−1^/1335.68 cm^−1^, respectively. D peak (1350.00 cm^−1^) was originated from a second-order process, relating to the structural defects or sp^3^ hybrid carbon atoms.^38^ The intensity ratio of D band to G band (*I*_D_/*I*_G_) of GO, 200-P-RGO, and 300-P-RGO samples are 0.969, 0.897, and 0.876 respectively. It suggested that the crystallization quality of the graphene became better through the combustion treatment. Under HT-200 °C condition, the explosion was more violent which produce more defects. This argument combined with the *I*_D_/*I*_G_ data above explained that the 300-P-RGO sample had a better crystallization quality than the 200-P-RGO sample.

The XPS were performed to analyse the degree of reduction of GO after the thermal treatment. Fig. 6c–e show the C 1s peaks of GO and P-RGO samples (HT-300 °C and 200 °C). The spectrum of GO was featured by the peaks at 284.8, 286.8, 287.8 eV, which were respectively corresponding to C–C/C=C, C–O, and C=O bonds.^39^ The spectrums of P-RGO (300 °C and 200 °C) have a more prominent peak of C–C/C=C bond at 284.8 eV and relatively weaker peaks of C–O and C=O bonds, suggesting the removal of most oxygenated groups of GO by the combustion treatment. The molar ratios of C atom to O atom were 2.03 (GO), 6.35 (300-P-RGO) and 8.35 (200-P-RGO) respectively. This result not only explained the reduction of GO but also provided further evidence that 200-P-RGO had a stronger reduction than 300-P-RGO. Due to the violent explosion at HT-200 °C, more pores with diameter larger than 2 nm were produced. Combined with the results of BJH measurements, the fact that 200-P-RGO had a smaller SSA than 300-P-RGO could be explained.

The mechanism for producing P-RGO could be explained as follows. In the preparation process of GO, the intensive oxidation not only introduced a significant number of oxygenated functional groups (–OH, –C=O and epoxide groups) onto graphite sheets, but also generated lots of defects on the sheets. In the process of thermal treatment, when the GOA sample contacted the preheated hot plate, the position of the defect, oxygen-containing functional group and edge sites of GO sheets first got to the fire point because of the instability of the carbon atoms in these positions, and the sample started to burn with bright flame (Fig. 2), the whole process causing the removal of oxygen groups by formation of carbonaceous species (CO_2_, CO) thus creating defects in the form of etching holes within graphene basal plane, resulting in the reduction of GO. As shown in the FTIR spectra in Fig. 6(a), the oxygen-containing functional groups mostly are –OH, C–O–C, C=O, –COOH, and the edge sites of GO is dominated by functional group –COOH. Abijit Ganguly *et al.*^40^ suggested, the activity of functional groups, from strong to weak, is: –COOH → C=O → C–O → –OH with the increase of temperature. However, the order of the reactivity of the defect and the functional group is still unclear. Furthermore, these fast out-diffusion gasses brought about a large pressure stronger than the van der Waals forces between the carbon atom layers, which could exfoliate the physically densely stacked layers. At the same time, the gasses would strike around the defect sites, thus creating additional pores on the sheets. On the other hand, the high temperature produced by combustion also offer heat for the adjacent GO to burn until the whole aerogel burnout. Overall, the process of combustion not only got the GO reduced, but also generated nanopores on the sheets. Thus nanoporous graphene with relatively large SSA was obtained.

**Fig. 7:**
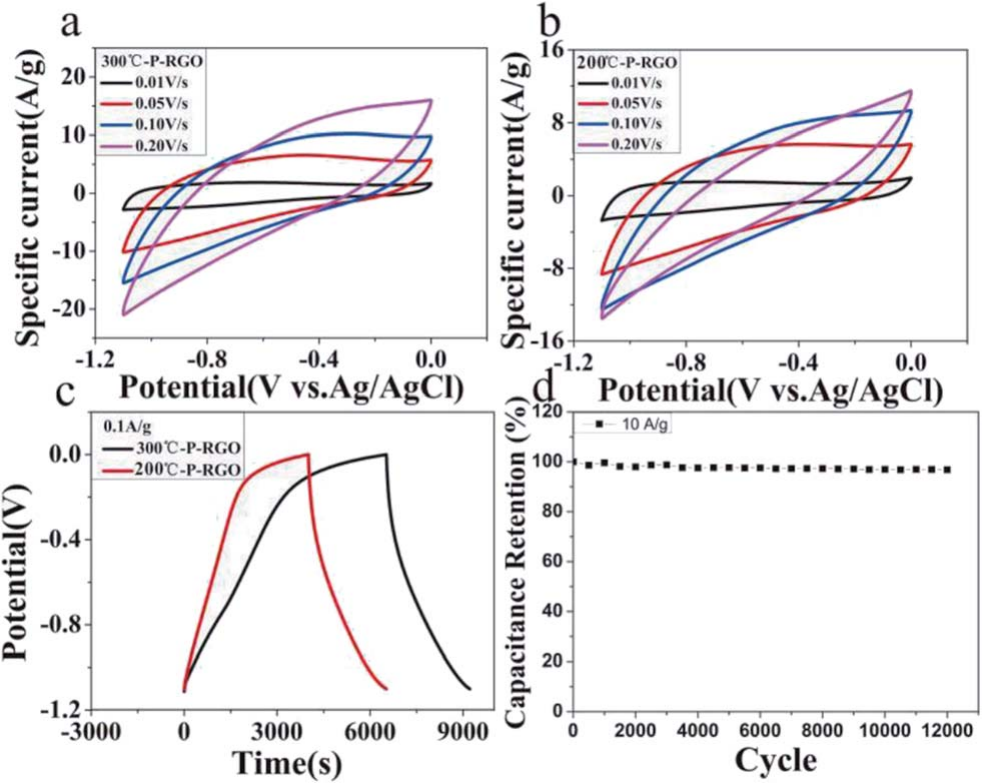
Electrochemical tests of P-RGO based supercapacitors: (a) CV graph of 300-P-RGO, (b) CV graph of 200-P-RGO, (c) GCD graph of 300-P-RGO and 200-P-RGO at the current density 0.1 A g^−1^, and (d) the capacitance retention rates for various cycle numbers up to 12 000 cycles at the current density 10A g^−1^ by the GCD test.

Here, the obtained P-RGO was utilized in supercapacitors. And, the electrochemical characteristics of P-RGO based supercapacitors were investigated. As shown in Fig. 7, the cyclic voltammetry (CV) and the galvanostatic charge/discharge (GCD) graphs of 300-P-RGO and 200-P-RGO samples are presented to evaluate the electrochemical performance of the electrodes in a three-electrode system. Fig. 7a and b show the CV curves measured at the scan rates of 10–500 mV s^−1^. The shapes of CV curves without any redox peaks demonstrated an excellent capacitance behavior and fast diffusion of electrolyte ions into the electrode.^41^ The GCD graph (Fig. 7c) shows the charge/discharge properties of the supercapacitor's electrode, in which we can see that P-RGO (HT-300 °C and 200 °C) based supercapacitors almost had the same discharge time. The specific capacitance (SC) was calculated by the formula *C*_g_ = (*It*)/(Δ*Vm*). At the scan rate 0.1 A g^−1^, 300-P-RGO based supercapacitor had an SC of 245 F g^−1^, and 200-P-RGO based supercapacitor had a SC of 229 F g^−1^. The SC data were shown in Table 2, for contrast, we also listed some previously reported results of porous graphene. In the table, the SC data of P-RGO based supercapacitor are relatively higher, which imply better electrochemical properties of P-RGO. The GCD curves exhibit unsymmetrical shapes,^42,43^ indicating that the electrode of the supercapacitor prepared by P-RGO has less reversibility, and the shapes of charge curves suggest that the resistance of the electrodes is large and pseudocapacitive behavior may be exist. By comparison, the reversibility of the electrode prepared by 200-P-RGO is better than that prepared by 300-P-RGO, and in the case of no significant difference in specific capacitance between them, 200-P-RGO seems to be more advantageous in the supercapacitor application. Fig. 7d reveals that the retention rate of the specific capacitance of 300-P-RGO based supercapacitor was higher than 96.8% after 12 000 cycles. High retention rate suggests excellent stability of this material and thus a promising application to super-capacitors, batteries and other energy storage/release devices.

**Table 2:** Specific capacitance

Material	Electrolyte	Capacitance (F g^−1^)
Activated porous graphene nanoribbons^44^	EMIM·BF_4_	130
Activated MEGO^21^	BMIM·F_4_/AN	166
Activated MEGO^45^	BMIM·F_4_/AN	172
Porous graphene/activated carbon composite^46^	6 M H_2_SO_4_	210
Activated 3D porous graphene^47^	TEA·BF_4_/AN	202
Porous graphene-like nanosheets^48^	6 M KOH	276
300-P-RGO (this work)	6 M KOH	245
200-P-RGO (this work)	6 M KOH	229

At last, it would be worth to mention the production yield of the P-RGO was more than 77.0% (through the whole process from the graphite powder to the P-RGO). Thus, this method might be applied to industrial production.

## 3. Experiment

### 3.1. Preparation of graphene oxide aerogel & paper (GOA, GOP)

All the reagents used in the experiment were analytical grade. Graphene oxide (GO) was synthesized by a modified Hummers method^49–51^ from the natural graphite powder. Then, aqueous suspension of GO with concentration of 10 mg ml^−1^ was prepared.

The GO suspension was put into a sample box in the refrigerator, in which it was frozen. Then the sample was moved into the cold well of the freeze dryer which had been cooled down to −50 °C for drying. After several hours' freeze-drying, GOA was prepared, of which the color was brown. As to the GOP preparation, the suspension was uniformly coated onto the glass plates and dried at 55 °C in the constant temperature drying box. About 6 hours later, the dried GO layer was pealed from the glass plate, which looked like a paper and so is named GOP.

### 3.2. Preparation of nano-porous reduced graphene oxide (P-RGO)

Under the atmospheric environment, the obtained GOA/GOP was put onto the hot plate preheated to the temperature 300 °C (HT-300 °C). Immediately, a flame was observed from the contacting point between the GOA/GOP and the hot plate, and quickly spread to the whole GOA/GOP. After about 0.3 s, the flame disappeared, the black and fluffy P-RGO was obtained. The similar experiments at HT-200 °C, 400 °C, 500 °C were also carried out. It is noticed that violent explosion produced by the combustion could be observed when the experiment was carried out at HT-200 °C. However, nearly no explosion could be seen in the combustion at HT-300 °C, 400 °C, 500 °C.

The obtained GO and P-RGO were characterized by field-emission scanning electron microscopy (SEM, JSM-6700F, JEOL, Japan), field emission transmission electron microscopy (FETEM, JEM-2100F), FT-IR spectroscopy (Nicolet 8700 FT-IR spectrometer), Raman microscopy (Horiba Jobin Yvon LabRAM HR-800), X-ray photoelectron spectroscopy (XPS) and MFA-140 (in this measurement, the specific surface area value was determined by the Brunauer–Emmett–Teller (BET) method and the pore size distribution was calculated with the Barrett–Joyner–Halenda (BJH) method).

### 3.3. Preparation and electrochemical measurement of P-RGO based supercapacitors

Firstly, P-RGO, acetylene black and polyvinylidene fluoride (PVDF) with a mass ratio of 40 : 5 : 5 were mixed in the NMP, forming a slurry. Then, a nickel foam (1.0 × 0.5 cm^2^) was immersed into the slurry for absorbing P-RGO and afterword dried at 60 °C for about 24 h in the constant temperature drying oven. The P-RGO contained nickel foam served as an electrode of the supercapacitor. Each electrode contained about 4.5 mg active materials.

A three-electrode system was used here for measuring the electrochemical properties: the P-RGO contained nickel foam (obtained at HT: 300 °C or 200 °C) worked as the working electrode, a platinum foil worked as the counter electrode, and an Ag/AgCl electrode worked as the reference electrode. The 6 M KOH aqueous solution was used as the electrolyte. CV and GCD tests were performed under the working potential ranging from −1.1 to 0 V (*vs.* Ag/AgCl) with different scan rates (10, 50, 100, 200, and 500 mV s^−1^) (for CV), and current densities (0.1, 0.2, 0.5, 1.0, 5.0, and 10.0 A g^−1^) (for GCD). The equation for calculating the specific capacitance (*C*_g_, F g^−1^) was *C*_g_ = (*It*)/(Δ*Vm*), where *I* (A) was the charge/discharge current, *t* (s) was the discharge time, Δ*V* was the potential range, and *m* (g) was the weight of the active materials contained in the electrode.

The characterization of the fabricated supercapacitor were performed on electrochemical workstation (CHI 760E).

## 4. Conclusions

We presented a combustion method for producing nanoporous graphene from graphene oxide, and the application of the obtained material in supercapacitors. The obtained P-RGO samples had nanopores with diameter mainly ranging around 0.4–2.0 nm and SSA as large as 536 m^2^ g^−1^. The P-RGO based supercapacitor exhibited a large specific capacitance 245 F g^−1^ at 0.1 A g^−1^ and a more than 96.8% retention rate after 12 000 cycles with respect to the initial specific capacitance at a scan rate 10 A g^−1^. Compared to other methods for producing porous graphene, our approach was more efficiency, rapid, environment friendly and low-cost, and could be implemented at relatively low HT-200 °C. Furthermore, this method had a high production yield of 77.0%, implying a possible industrial production. The P-RGO made by our method might have a significant potential in application to gas adsorption/storage, energy storage, dye adsorption and even could be used in compounding with other materials for getting new properties. This work shows the feasibility of the combustion method for producing nanoporous graphene, but the technique is not mature enough. There are still many problems need to be solved in producing the material in industrial production, also in applications.

## Conflicts of interest

There are no conflicts to declare.

## Supplementary Material

RA-008-C7RA13568H-s001

RA-008-C7RA13568H-s002

RA-008-C7RA13568H-s003

RA-008-C7RA13568H-s004
