# Supplementary material for: A combustion method to synthesize nanoporous graphene
Source: RSC Adv. 2018 Mar 5;8(17):9320–6. doi: 10.1039/c7ra13568h (PMC9078665; doi:10.1039/c7ra13568h)
Supplement: RA-008-C7RA13568H-s001 [file RA-008-C7RA13568H-s001.pdf]

Electronic Supplementary Material (ESI) for RSC Advances.
This journal is © The Royal Society of Chemistry 2018

Supporting Online Material for
A combustion method for synthesizing nanoporous graphene

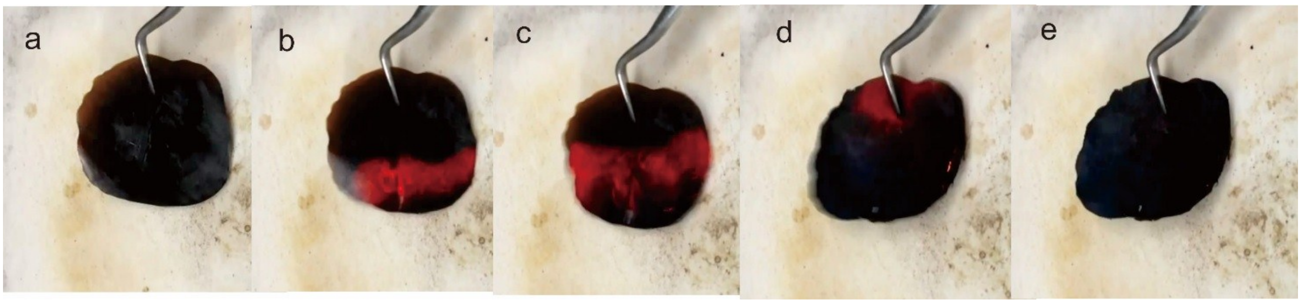

Fig. S.I.1- Optical photos of the combustion and expansion process of GOP at 300 ˚C. Graph (a) is the brown color GOP before contacting the hot heating plate, graph (b)-(d) are the process of combustion, graph (e) is the gained black fluffy porous RGO.
